# A proteotranscriptomic approach to dissect the molecular landscape of human retinoblastoma

**DOI:** 10.3389/fonc.2025.1571702

**Published:** 2025-05-06

**Authors:** Julian Wolf, Rozina Ida Hajdu, Stefaniya Boneva, Ira Godbole, Lucas Stürzbecher, Claudia Auw-Haedrich, Wolf A. Lagrèze, Hansjürgen Agostini, Thomas Reinhard, Stefan Tholen, Oliver Schilling, Günther Schlunck, Bertram Bengsch, Clemens Lange

**Affiliations:** ^1^ Eye Center, Medical Center, Faculty of Medicine, University of Freiburg, Freiburg, Germany; ^2^ Clinic for Internal Medicine II, Gastroenterology, Hepatology, Endocrinology, and Infectious Disease, Medical Center, Faculty of Medicine, University of Freiburg, Freiburg, Germany; ^3^ Institute of Surgical Pathology, Medical Center, Faculty of Medicine, University of Freiburg, Freiburg, Germany; ^4^ Ophtha-Lab, Department of Ophthalmology, St. Franziskus Hospital, Münster, Germany

**Keywords:** retinoblastoma, transcriptomics, proteomics, IMC, proteotranscriptomics, translational medicine

## Abstract

**Background:**

Retinoblastoma is a rare pediatric eye cancer caused by mutations in the RB1 gene, which regulates retinal cell growth. Early detection and treatment are critical for preventing vision loss and improving survival outcomes. This study aimed to perform an integrated proteotranscriptomic characterization of human retinoblastoma to provide a deeper understanding of disease biology and to identify novel therapeutic targets.

**Methods:**

Paired tumor and adjacent retinal tissue samples were dissected from seven eyes. RNA sequencing and liquid chromatography-mass spectrometry were performed on the same samples. The spatially resolved cellular landscape was assessed using Imaging Mass Cytometry (IMC).

**Results:**

The correlation between RNA and protein level was moderate with variations across different pathways, underscoring the value of an integrated proteotranscriptomic approach. IMC identified more than 67,000 single cells in 11 distinct clusters, including antigen presenting cells, T cells, stroma cells, vascular cells and two clusters of proliferating and CD44/c-Myc positive tumor cells. Antigen presenting cells expressed higher levels of CD68 in retinoblastoma compared to controls.

**Conclusions:**

CD44+ and high-c-Myc-expressing tumor cells may represent cancer stem cells with possible involvement in metastasis, warranting further validation. Our multilayered approach could pave the way for enhanced molecular assessments and novel targeted therapies for human retinoblastoma.

## Introduction

Retinoblastoma is the most common intraocular malignancy in childhood, affecting about 8,000 children globally each year (1, 2). Despite its low incidence, retinoblastoma has provided crucial insights into human tumorigenesis, notably the two-hit mechanism of tumor-suppressor gene inactivation, which involves a mutation in both alleles of the *RB1* gene (3). Early detection of retinoblastoma leads to favorable treatment outcomes, but advanced tumors often necessitate eye removal and are associated with a high risk of recurrence and life-threatening metastasis. Additionally, chemoresistant tumors and the adverse effects of systemic or local chemotherapy underscore the urgent need for novel therapeutic approaches (2). Transcriptional profiling has shed light on disease mechanisms and led to the identification of promising therapeutic targets, such as MDM2 antagonists and MYCN inhibitors (2, 4). However, most studies focus on gene expression profiling without accounting for proteomic changes. Since proteins are the main effectors of disease and targets for most drugs, integrating proteomics and transcriptomics may provide additional information for a deeper understanding of retinoblastoma biology, as previously demonstrated for other cancer types (5–7).

In this study, we conducted a proteotranscriptomic analysis of human retinoblastoma by collecting paired tumor and adjacent retinal tissue samples serving as controls. The global transcriptome and proteome were characterized using RNA sequencing and liquid chromatography-mass spectrometry (LC-MS/MS). Additionally, we performed Imaging Mass Cytometry (IMC) on the same tissue to spatially resolve their cellular landscape. Our findings demonstrate that integrating multiple omics technologies offers deeper insights into retinoblastoma biology, surpassing the limitations of single-omics approaches.

## Methods

### Patients and collected samples

This study retrospectively analyzed seven eyeballs from seven children with retinoblastoma, who underwent enucleation at the Eye Center of the University of Freiburg between 1991 and 2000 (**Supplementary Table S1**). Two of the seven patients had bilateral retinoblastoma (**Supplementary Table S1**) and in these cases, the enucleated eye was analyzed in our study. In six of the seven patients, the tumor was initially unilateral and intraconal without evidence of extraocular invasion or metastasis, whereas no staging information was available for the seventh patient (patient 1, **Supplementary Table S1**). Patients 6 and 7 developed a tumor on the fellow eye 11 and 6 months after enucleation, which were treated externally. For all cases, no information regarding chemotherapy or radiotherapy was available. Parents of all seven children were suggested to attend genetic consultation and testing, which was performed externally. We only had access to the results of the genetic testing for patient 2 (**Supplementary Table S1**). Parents of patient 7 denied genetic testing. All procedures were conducted in accordance with relevant guidelines and regulations and ethics approval was granted by the Institutional Review Board (IRB) of the Albert-Ludwigs-University Freiburg (approval number 21-1246). Informed consent was obtained from the subjects. In some cases, patients could no longer be reached because the surgery had been performed several years ago. In those cases, the need for informed consent was waived by the IRB of the Albert-Ludwigs-University Freiburg.

### Formalin fixation and paraffin embedding

Formalin fixation and paraffin embedding (FFPE) of tissue samples was performed immediately after surgery according to routine protocols, as previously described (8, 9). Briefly, samples were fixed immediately after surgery in 4% formalin for 48 hours, dehydrated in alcohol and processed for paraffin embedding.

### Tissue dissection

Paired retinoblastoma and adjacent retinal tissue samples were collected from seven enucleated retinoblastoma eyes. Histopathological evaluation by at least two experienced ophthalmic pathologists confirmed the presence of retinoblastoma in all seven cases and adjacent retinal tissue in five cases. In the remaining two eyes, normal retinal tissue could not be isolated. For RNA sequencing and liquid chromatography-mass spectrometry (LC-MS/MS), tumor and control tissues were carefully isolated before analysis using a macrodissection protocol (10) to remove unwanted tissue prior to analysis. Briefly, 10 µm tissue sections were obtained from FFPE tissue blocks and the first and last sections were processed for hematoxylin and eosin (H&E) staining. Sections were reviewed by an experienced ophthalmic pathologist to mark the regions of tumor and microscopically unaffected adjacent retinal tissue. The first and last sections were imaged using a Hamamatsu NanoZoomer S60 (Hamamatsu Photonics, Herrsching, Germany) and the area of tumor and retinal tissue were determined in mm^2^ using the company’s NPD.viewer software. Based on the size of the region of interest (ROI) in mm^2^, the minimum number of 10µm sections were calculated to reach a tissue volume of at least 0.7 mm^3^ for downstream analysis. The last tissue section (H&E) was used to confirm that tumor and retinal tissue were still present and to confirm similar ROI size. If the ROI size was smaller in the last tissue section compared to the first section, the smallest value was used to calculate the minimum number of tissue sections. The marked H&E sections were used to guide resection of tumor and control tissue from the calculated number of serial unstained sections from the same tissue block. For transcriptomics and proteomics downstream analyses, we each obtained a mean number of 2.6 tissue sections (range: 1 – 6) for tumor tissue and 28.8 sections (range: 10 – 70) for adjacent retinal control tissue. For Imaging Mass Cytometry (IMC), tissue sections were obtained from the same FFPE blocks as described below and ROIs were selected directly at the IMC instrument before capturing the data.

### Transcriptomics using RNA sequencing

#### RNA isolation

FFPE samples were shipped in tubes at room temperature to the sequencing provider (GenXPro, Frankfurt, Germany) and total RNA was isolated using the Quick-RNA FFPE Kit (Zymo Research, Irvine, CA, USA), as previously described (11). Following a DNAse I digestion using the Baseline-ZERO kit (Epicentre, Illumina, San Diego, CA, USA), the RNA concentration was measured with the Qubit RNA HS Assay Kit on a Qubit Fluorometer (Life Technologies, Carlsbad, CA, USA). The RNA quality was determined with the RNA Pico Sensitivity Assay on a LabChip GXII Touch (PerkinElmer, Waltham, MA, USA).

#### RNA sequencing

RNA sequencing was performed using massive analysis of cDNA ends (MACE), a 3’ RNA sequencing method, as previously described (11). We recently demonstrated that MACE allows sequencing of FFPE samples with high accuracy, even after more than 10 years of storage (12). Barcoded libraries comprising unique molecule identifiers were sequenced on the NextSeq 500 (Illumina, San Diego, CA, USA) with 1 × 75 bp. PCR bias was removed using unique molecular identifiers.

#### Data analysis

Sequencing data (fastq files) were uploaded to and analyzed on the Galaxy web platform (usegalaxy.eu) (13), as previously described (14). Quality control was performed with *FastQC Galaxy Version 0.73* (http://www.bioinformatics.babraham.ac.uk/projects/fastqc/ last access on 11/05/2022). Reads were mapped to the human reference genome (Gencode, release 42, hg38) with *RNA STAR Galaxy Version 2.7.8a* (15) with default parameters using the Gencode annotation file (Gencode, release 42, https://www.gencodegenes.org/human/releases.html). Reads mapped to the human reference genome were counted using *featureCounts Galaxy Version 2.0.1* (16) with default parameters using the aforementioned annotation file. The output of featureCounts was imported to RStudio (version 2024.04.2 + 764, R version 4.4.1). Gene symbols and gene types were determined based on ENSEMBL release 108 (Human genes, GRCh38.p12, download on 11/05/2022) (17). Principal Component Analysis (PCA) was applied to assess unsupervised clustering and to check for potential batch effects (18). Differential gene expression was analyzed using the R package DESeq2 Version 1.44.0 (18) with default parameters (Benjamini-Hochberg adjusted p-values). Transcripts with log2 fold change (log2 FC) > 1 or < -1 and adjusted *p*-value < 0.05 were considered as differentially expressed genes (DEG). Heatmaps were created with the R package *ComplexHeatmap 2.20.0* (19). Other data visualization was performed using the *ggplot2* package (20). Gene enrichment analysis and its visualization were conducted using the R package *clusterProfiler 4.12.0* (21). Cell type enrichment analysis was performed using xCell (22). The tool uses sequencing-derived transcriptomic signatures of 64 distinct immune and stroma cell types to estimate the relative contributions of these cells to a bulk RNA transcriptome. Transcripts per million were calculated as an input for the analysis based on the output of featureCounts (assigned reads and feature length), as previously described (23). xCell enrichment scores were compared between different groups using the Mann–Whitney U test.

### Proteomics using liquid chromatography-mass spectrometry

#### Sample preparation

Prior to macrodissection, all sections were deparaffinized in xylene and rehydrated passing a series of alcohol solutions in descending concentration. Sections were stained with haemalaun for 10 seconds and macrodissection was performed as described above. The isolated tissue was stored in tubes at -80°C until downstream analysis. 100 µl of an aqueous buffer containing 4% SDS in 0.1M HEPES were added into each tube. Proteins were extracted by sonication using a Bioruptor device (Diagenode, Liège, Belgium) and incubating the samples in a thermoshaker at 95°C for 2h, and a second sonication step using the Bioruptor. Samples were centrifuged at 13000g for 8 min and the supernatants used in the following steps. Proteins were reduced using 5 mM tris (2-carboxyethyl) phosphine hydrochloride (TCEP) (Sigma; 75259) for 10 min at 95°C and alkylated using 10 mM 2-iodoacetamide (Sigma; I1149) for 20 min at room temperature in the dark. The following steps were performed using S-Trap micro filters (Protifi, Huntington, NY) following the manufacturer’s procedure. Briefly, first a final concentration of 1.2% phosphoric acid and then six volumes of binding buffer (90% methanol; 100 mM TEAB; pH 7.1) were added. After gentle mixing, the protein solution was loaded to a S-Trap filter and spun at 2000 rpm for 0.5–1 min. The filter was washed three times using 150 μL of binding buffer. Sequencing-grade trypsin (Promega, 1:25 enzyme:protein ratio) diluted in 20µl digestion buffer (50 mM TEAB) were added into the filter and digested at 47°C for 1 h. To elute peptides, three buffers were applied stepwise: a) 40 μL 50 mM TEAB, b) 40µl 0.2% formic acid in H2O, and c) 50% acetonitrile and 0.2% formic acid in H2O. The peptide solutions were combined and dried in a SpeedVac.

The peptide concentration was determined using BCA and 25 µg of each sample was transferred to a fresh microreaction tube. 0.15 M HEPES pH 8.0 was added. Samples were labeled using TMT-16-plex (Thermo Scientific) (24). Afterwards, samples were combined and ~100 µg of protein were fractionated by high pH reversed phase chromatography [XBridge C18 column, 150 mm × 1 mm column containing 3.5 µm particles (Waters)]. An increasing linear gradient of acetonitrile from 10 to 45% over 45 min at a flowrate of 42 µl/min was applied using an Agilent 1100 HPLC system. 36 fractions were collected and concatenated into 10 fractions, which were vacuum-concentrated until dryness and stored at − 80°C until LC–MS/MS analysis.

#### Data acquisition

For LC-MS/MS measurements, 800 ng of peptides were analyzed on a Q-Exactive Plus mass spectrometer (Thermo Scientific, San Jose, CA) coupled to an EASY-nLCTM 1000 UHPLC system (Thermo Scientific). The column setup consisted of an Acclaim™ PepMap™ 100 C18 column (Thermo Fisher Scientific, Cat. No. 164946) and a 200 cm µPac GEN1 analytical column (PharmaFluidics, 55250315018210) coupled to a Nanospray FlexTM ion source (Thermo Scientific, ES071) and a fused silica emitter (MS Wil, TIP1002005-5). For peptide separation, a linear gradient of increasing buffer B (0.1% formic acid in 80% acetonitrile, Fluka) was applied, ranging from 5 to 50% buffer B over the first 80 min and from 50 to 100% buffer B in the subsequent 40 min (120 min separating gradient length). Peptides were analyzed in data dependent acquisition mode (DDA). Survey scans were performed at 70,000 resolution, an AGC target of 3e6 and a maximum injection time of 50 ms followed by targeting the top 10 precursor ions for fragmentation scans at 17,500 resolution with 1.6 m/z isolation windows, an NCE of 30 and a dynamic exclusion time of 35 s. For all MS2 scans the intensity threshold was set to 1e5, the AGC to 1e4 and the maximum injection time to 80 ms.

#### Data analysis

Raw data were analyzed with MaxQuant (v 1.6.14.0) with the built-in Andromeda peptide search engine (25). The false discovery rate (FDR) at both the protein and peptide level was set to 1%. Two missed cleavage sites were allowed, no variable modifications, carbamidomethylation of cysteines as fixed modification, and 16 plex TMT as isobaric label. The Human-EBI-reference database was downloaded from https://www.ebi.ac.uk/ on Jan 9th 2020. Only unique peptides were used for quantification.

Statistical analysis was performed using the MSstatsTMT package (v. 1.8.2) in R (v. 4.0.3). Subsequently, protein intensities were log2 transformed. To identify differentially expressed proteins, we used the limma package (v. 3.46.0) in R using the “robust” method. P-values were adjusted using the Benjamini-Hochberg procedure.

### Integration of transcriptomics and proteomics data

Processed transcriptomics and proteomics data (see above) were integrated in R. Before joining, Gene symbols were filtered for approved symbols using the HGNC (Human Genome Organization Gene Nomenclature Committee) database (26). For each symbol where both protein and mRNA data were available, the log2FC between retinoblastoma and controls were plotted between transcriptomics and proteomics using ggplot2. Gene enrichment analysis and its visualization were done using the R package *clusterProfiler 4.12.0* (21). Briefly, differentially expressed genes and proteins were determined using the following criteria: log2FC > 0.58 or < -0.58 and adjusted p-value < 0.05. A functionally grouped network of enriched Gene Ontology (GO) biological processes was generated using the emapplot function of the *clusterProfiler* R package.

### Imaging mass cytometry

#### Tissue preparation

For Imaging Mass Cytometry (IMC), 6 µm tissue sections were obtained from the same seven FFPE blocks (**Supplementary Table S1**), mounted on slides (SuperFrost Plus, Thermo Scientific, USA) and dried at 60°C in an oven for 90 minutes, as previously described (27). To prevent oxidative degradation, the sections were processed immediately for staining. Sections were deparaffinized in xylene and rehydrated passing through a series of alcohol solutions in descending concentration. Heat-induced antigen retrieval was performed using DAKO EnvisionFlex target retrieval solution (high pH, Agilent Technologies) at 95°C for 30 min in a pressure cooker. Subsequently, slides were blocked in 3% BSA in tris-buffered saline (TBS) for 60 minutes at room temperature and a specifically compiled panel of antibodies (Fluidigm) was used to stain the sections. A complete list of antibodies, conjugated metals, and applied concentrations used in this study is shown in **Supplementary Table S2**. Antibodies were mixed and the cocktail was applied to the sections and incubated overnight at 4°C in a hydration chamber. After washing with TBS, an iridium-intercalator solution was applied to the slides and incubated for 5 minutes at room temperature. Slides were then dried at room temperature for 30 minutes and stored in a box until data acquisition, which was performed in the following days.

#### Image acquisition

Images were acquired using the Hyperion Imaging System (Fluidigm), with instrument tuning performed according to manufacturer’s instructions. Regions of interest were identified by dark-field microscopy before acquisition. Tissue sections were laser-ablated spot-by-spot at 200 Hz at laser power 2 resulting in a pixel size/resolution of 1 μm^2^. Several 1500 μm^2^ images per sample were produced. Raw data were processed using the CyTOF software v7.0 (Fluidigm). Images were reviewed using the MCD Viewer v1.0.560.6 (Fluidigm).

#### Cell segmentation

Imaging Mass Cytometry (IMC) data was processed as previously described (27). Briefly, mcd files were converted into TIFF image stacks following a Python script adapted from the ImcSegmentationPipeline of the Bodenmiller group (28). Segmentation probabilities were generated using ilastik (version 1.3.2) to designate nuclei, cytoplasm and background fractions and the probability maps were imported into CellProfiler (Version 3.1.8) (29) to extract single-cell information. Data were further processed using histoCat (Version 1.76) (30) to calculate the mean marker intensity of pixels.

#### Data analysis

IMC data were imported in R studio (version 2024.04.2 + 764, R version 4.4.1) and analyzed according to the ImcDataAnalysis Pipeline of the Bodenmiller group (last access 04/2022) (28). Briefly, single-cell data, including cell identifiers, image identifiers, sample metadata, spatial information, neighbors and mean pixel intensities per cell and channel were combined into a SpatialExperiment object. Two samples had to be removed from downstream analysis (Retinoblastoma_S1 and Control_S2, **Supplementary Table S1**), because almost all markers showed no or unspecific staining in these samples. In addition, the markers FoxP3 and Arginase-1 were excluded from further downstream analysis due to unspecific staining. All other markers demonstrated satisfactory staining and were retained for further analysis. Uniform Manifold Approximation and Projection (UMAP) was performed for dimension reduction. The fastMNN function of the batchelor package was applied to correct batch effects and integrate cells between patients (31). The shared nearest neighbor clustering approach was used to identify clusters of cells (k = 45). Cell clusters were annotated based on known cell type markers.

## Results

### Samples for multi-omics analyses

We collected paired retinoblastoma and adjacent retinal tissue samples from seven patients with retinoblastoma who underwent enucleation at our institution (**Supplementary Table S1**). Histopathology confirmed retinoblastoma in all seven cases and adjacent retinal tissue in five cases (**Supplementary Figure S1**). The patients had a mean age of 2.0 years (range 0.1 – 5.1) and three patients were female (42.9%). For each of the 12 samples, we determined the global transcriptome, proteome, and the spatially resolved cellular landscape, resulting in a total of 34 high-resolution molecular datasets (**Supplementary Table S1**).

### Transcriptional profile of human retinoblastoma

**Figure 1:**
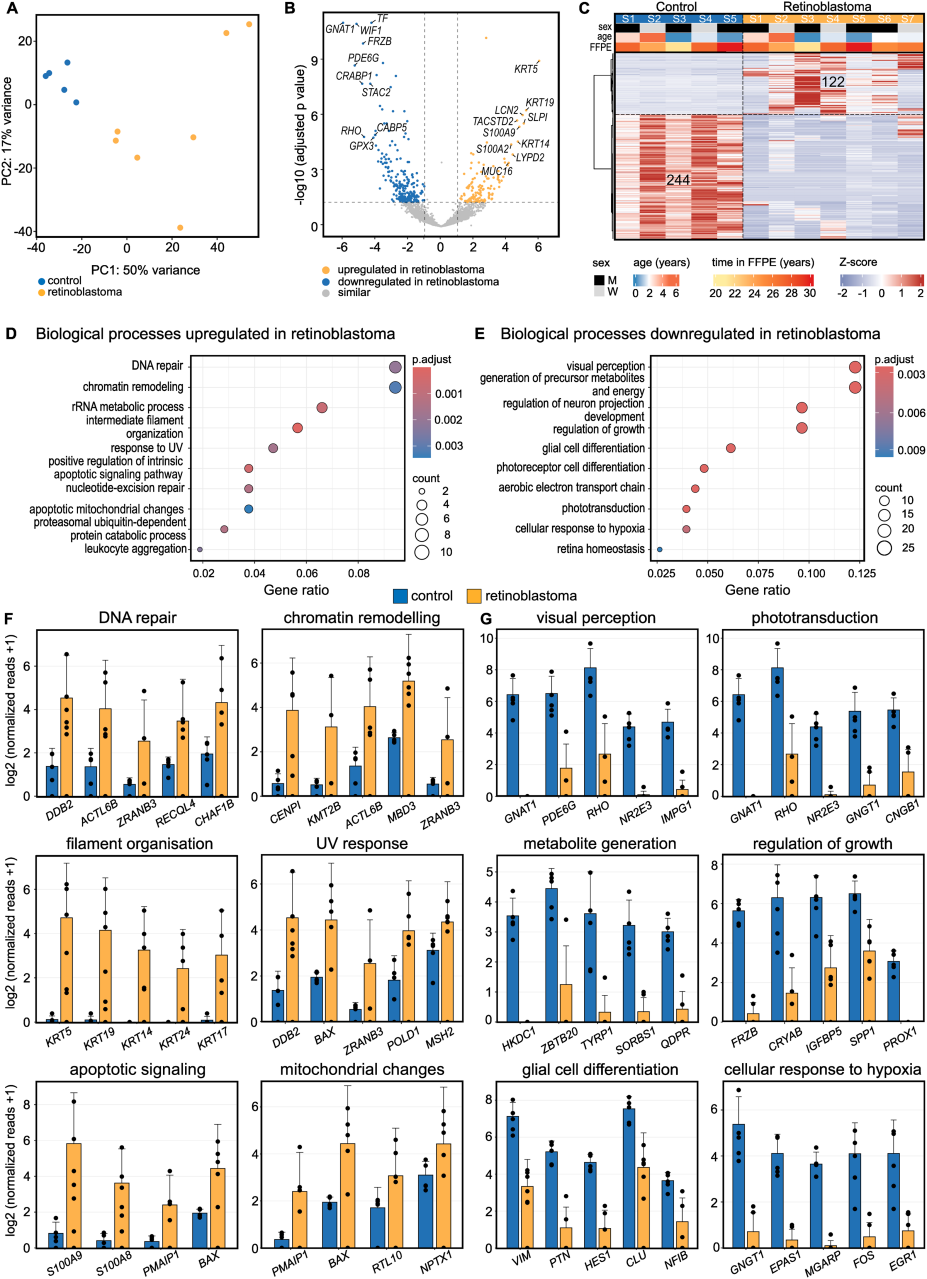
Transcriptional profile of human retinoblastoma. **(A)**: Unsupervised clustering using Principal Component Analysis (PCA). Each dot represents one sample. **(B)**: Volcano plot visualizing differentially expressed genes (DEG) between retinoblastoma and retinal control tissue. Each dot represents one gene. The top ten DEG of both groups are labeled. **(C)**: Heatmap visualizing DEG between retinoblastoma and retinal control tissue (Definition of DEG: log2FC > 1 or < -1 and adjusted p-value < 0.05). Basic demographic data is shown at the top. Each column represents one sample and each row one DEG. The number of DEG is given within the heatmap. The z-score represents a gene’s expression in relation to its mean expression by standard deviation units (red: upregulation, blue: downregulation). **(D, E)**: Gene ontology (GO) analysis of up- **(D)** and downregulated **(E)** genes in retinoblastoma. The top ten enriched biological processes are shown in the dot plots. The size of the dots corresponds to the number of associated genes (count). The adjusted p-value of each GO term is indicated by color. The gene ratio describes the ratio of the count to the number of all DEG. **(F, G)** The bar plots visualize gene expression of the top 5 DEG involved in 6 of the most significantly **(F)** up- or **(G)** downregulated biological processes. The height of the bar represents mean expression and the error bar corresponds to standard deviation. Each dot represents one sample.

First, we looked into the transcriptome of human retinoblastoma. Unsupervised cluster analysis using Principal Component Analysis (PCA) revealed clear distinctions between the transcriptional profiles of retinoblastoma and adjacent retinal control tissue (**Figure 1A**). Differential gene expression analysis identified 122 up- and 244 downregulated genes in retinoblastoma compared to controls (**Figures 1B, C**). Among the most upregulated genes were *KRT5*, *KRT19*, *LCN2*, *SLP1* and *S100A9, while GNAT1*, *PDE6G*, *WIF1*, *FRZB*, and *RHO* were among the top downregulated genes in retinoblastoma (**Figure 1B**). Gene ontology (GO) analysis revealed that the upregulated genes contributed most significantly to biological processes such as DNA repair (e.g. *DDB2* and *RECQL4*), chromatin remodeling (e.g. *CENPI* and *KMT2B*), intermediate filament organization (e.g. *KRT5* and *KRT19*), response to ultraviolet light (e.g. *DDB2* and *BAX*), and regulation of apoptosis (e.g. *PMAIP1* and *BAX*) (**Figures 1D, F**). The downregulated genes were most significantly linked to processes including visual perception (e.g. *GNAT1* and *RHO*), generation of precursor metabolites and energy (e.g. *HKDC1* and *SORBS1*), regulation of growth (e.g. *FRZB* and *PROX1*), glia cell differentiation (e.g. *VIM* and *PTN*) and response to hypoxia (e.g. *EPAS1* and *EGR1*) (**Figures 1E, G**). Gene Set Enrichment Analysis (GSEA) revealed similar processes being activated and suppressed in retinoblastoma (**Supplementary Figure S2**). These results underscore the distinct transcriptional landscapes of retinoblastoma and control tissue, highlighting key biological processes involved in human retinoblastoma.

### Proteotranscriptomic analysis of human retinoblastoma

**Figure 2:**
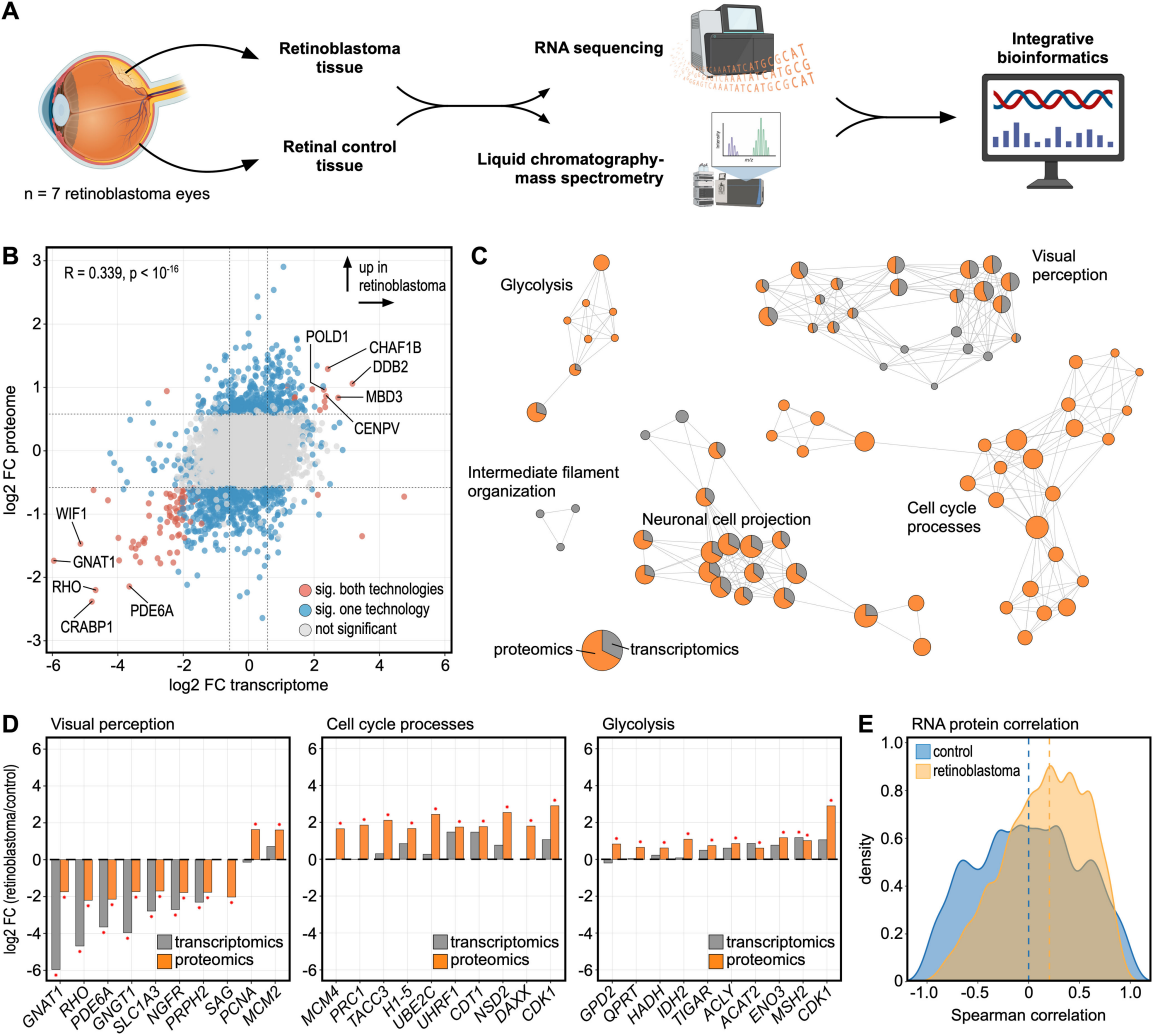
Proteotranscriptomic analysis of human retinoblastoma. **(A)** Experimental design. RNA-sequencing and liquid chromatography-mass spectrometry (LC–MS/MS) were applied to analyze the transcriptomic and proteomic profile of human retinoblastoma and retinal tissue specimens. **(B)** Comparison of the log2 fold change (FC) between retinoblastoma and retinal tissue on the transcriptomic (x-axis) and proteomic (y-axis) level. Each dot represents one gene/protein. Molecules with a significant difference between retinoblastoma and controls are shown in blue (proteomics or transcriptomics) or red (proteomics and transcriptomics). **(C)** Functionally grouped network analysis of enriched Gene ontology biological processes in which the up- or downregulated molecules were involved in. Enriched terms are visualized as nodes being linked based on the similarity of the factors associated with them. The node size represents the number of associated molecules. The pie charts visualize the percentage of molecules which were regulated on the transcriptomic (grey) or proteomic (orange) level. Each cluster is labeled with a representative term. **(D)** The top ten differentially expressed factors are shown for the three most affected groups of biological processes from **(C)** on the gene (grey) and protein (orange) level. Molecules with a significant change (adjusted p-value < 0.05) between retinoblastoma and controls are labeled with a red asterisk. **(E)** Density plots of Spearman’s correlation coefficients of RNA and protein levels of individual molecules between samples for retinoblastoma (yellow) and control retinal (blue) tissue. Dashed lines represent median Spearman correlation in each group.

To investigate how gene expression changes translate into proteomic changes in retinoblastoma, we conducted RNA sequencing and LC–MS/MS on both tumor and adjacent retinal tissue (**Figure 2A**). RNA sequencing revealed 15,716 expressed protein-coding genes. The peptides identified by LC-MS/MS were mapped to 4,535 proteins. Both protein and mRNA data were available for 4,173 protein-coding genes. Proteomics-only analysis demonstrated clear distinctions between retinoblastoma and control tissue (**Supplementary Figure S3**). Comparing the changes in retinoblastoma with control tissue revealed a moderate correlation between RNA and protein levels (Pearson’s R = 0.339, p < 10^-16^) (**Figure 2B**). The highest agreement between RNA and protein level was observed for DDB2, CHAF1B, and POLD1, which were upregulated in retinoblastoma, and RHO, PDE6A, and CRABP1, which were downregulated (**Figure 2B**). Network analysis of functionally grouped enriched GO biological processes highlighted that the differentially expressed factors were involved in five main clusters: regulation of cell cycle, glycolysis, neuronal cell projection, visual perception, and intermediate filament organization (**Figure 2C**). While clusters related to neuronal cell projection (e.g. GFAP and SLC1A3) and visual perception (e.g. RHO and PDE6A) showed comparable regulation at both RNA and protein levels, the clusters of cell cycle processes (e.g. MCM4 and PRC1) and glycolysis (e.g. GPD2 and ENO3) as well as intermediate filament organization (e.g. KRT5 and KRT19) were predominantly regulated at either the protein or RNA level, respectively (**Figures 2C, D**). Notably, the RNA protein correlation was significantly (p < 10^-16^) higher in retinoblastoma (median Spearman’s R = 0.204) compared to control tissue (median Spearman’s R = 0.0) (**Figure 2E**). These results indicate that key biological processes in retinoblastoma are mainly regulated at either the RNA or protein level, underscoring the importance of an integrated proteotranscriptomic approach.

### Highly multiplexed spatially resolved single-cell proteomics of human retinoblastoma

**Figure 3:**
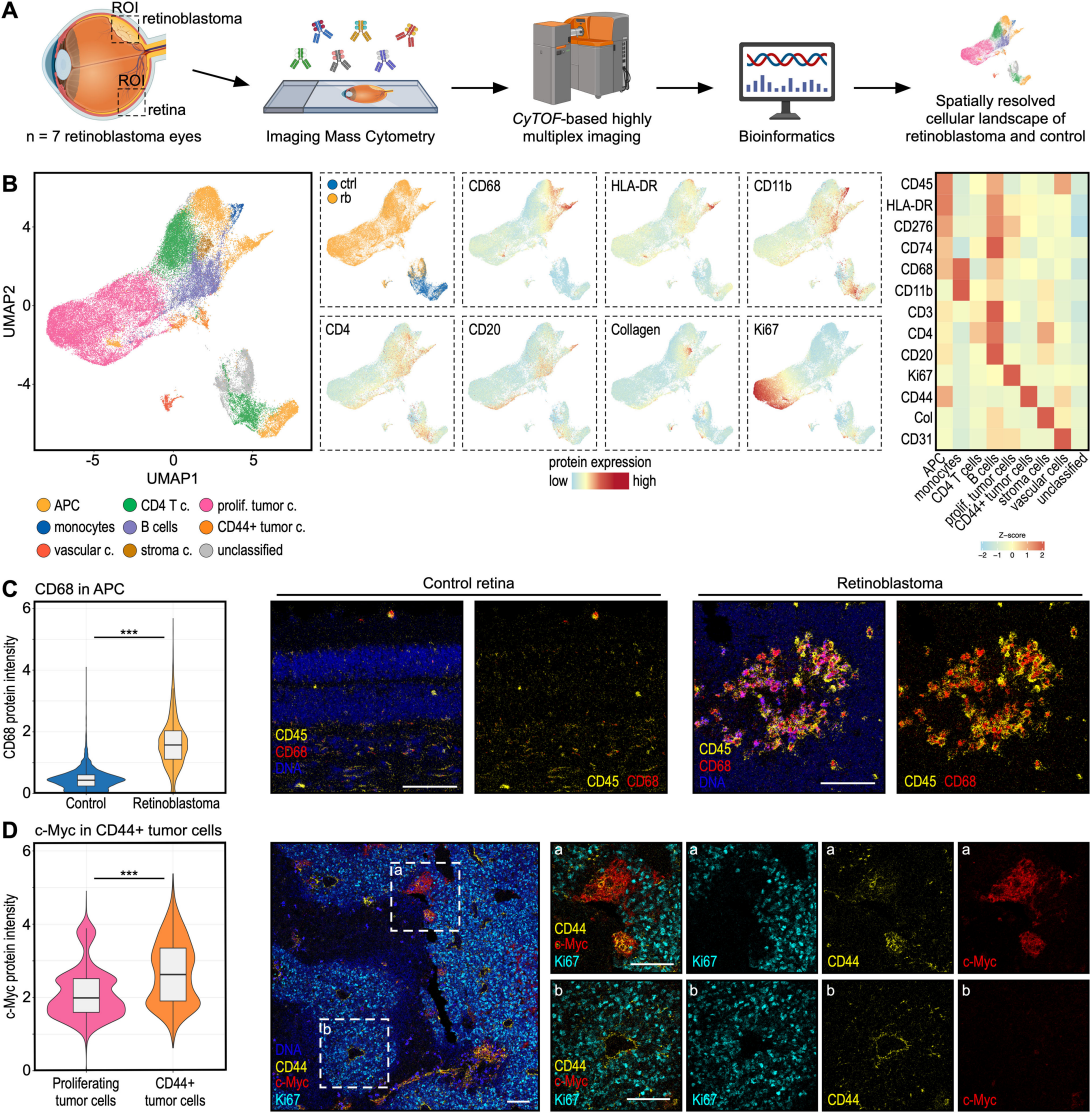
Highly multiplexed spatially resolved single-cell proteomics of human retinoblastoma. **(A)** Experimental workflow. Post-enucleation tissue slices from whole eyes of 7 patients with retinoblastoma were analyzed using Imaging Mass Cytometry (IMC). Microscopically unaffected retinal tissue from the same eye was used as control tissue. ROI: Region of interest. **(B)** Uniform Manifold Approximation and Projection (UMAP) visualization showing Phenograph clustering of 67,058 single cells in 7 human retinoblastoma (58,299 cells) and 5 human control retinal tissue samples (8,759 cells). Cell type annotation is shown by color (see legend). UMAPs showing diagnosis or relevant marker proteins. The heatmap on the right demonstrates average marker expression in each cell cluster. **(C)** Violin plots visualizing protein expression of CD68 in antigen presenting cells (APC) in retinoblastoma (yellow) and controls (blue). ***: p<0.001. Representative images are shown on the right. Magnifications of the dashed white boxes in the image on the left are shown on the right (marked with a or b). Each scale bar corresponds to 100 µm. **(D)** c-Myc protein expression between the two tumor cell clusters from **(B)**: proliferating tumor cells (pink) and CD44+ tumor cells (orange). ***: p<0.001. Representative images are shown on the right. Each scale bar corresponds to 100 µm.

To elucidate the cellular landscape of human retinoblastoma, we first performed transcriptome-based cell type deconvolution analysis using xCell (22), revealing high abundance of various immune cell types in retinoblastoma, most prominently CD4+ T helper cells, as well as pericytes and fibroblasts (**Supplementary Figure S4**). We next conducted highly multiplexed spatially resolved single-cell proteomics of tumor and adjacent retinal tissue using Imaging Mass Cytometry (IMC) (**Figure 3A**). We identified a total of 67,058 single cells in seven retinoblastoma (58,299 cells) and five control samples (8,759 cells). Unsupervised clustering analysis identified distinct cell clusters, including various immune cell types, such as antigen presenting cells (CD45+, HLA-DR+), monocytes (CD11b+), CD4+ T cells (CD4+) and B cells (CD20+), stroma cells (collagen+), vascular cells (CD31+), and tumor cells, including proliferating tumor cells (Ki67+) and CD44+ tumor cells (CD44+) (**Figure 3B**). Comparative analysis of antigen presenting cells between tumors and controls showed significantly higher CD68 protein expression compared to controls (**Figure 3C**).

Although CD68 is traditionally associated with phagocytosis, recent studies linked its increased expression to poor prognosis in several cancers, including hepatocellular, lung and other cancers (32). Finally, we compared the two main clusters of tumor cells: proliferating and CD44+ tumor cells, and found that c-Myc was one of the most significantly increased proteins in CD44+ tumor cells (**Figure 3D**). CD44 is a known marker of cancer stem cells (33), which are implicated in the initiation of metastatic disease, a process which has been associated with high c-Myc expression in other cancer types (34). These results indicate that CD44 and c-Myc expressing retinoblastoma cells may represent cancer stem cells. Future studies will be needed to investigate the functional and prognostic role of the identified cell population in human retinoblastoma.

## Discussion

Changes in gene expression do not always translate into proteomic changes (5–7, 35). To address this, we combined high-resolution proteomics and transcriptomics from the exact same samples to provide a comprehensive proteotranscriptomic analysis of human retinoblastoma. Our results revealed only a moderate correlation (Pearson’s R = 0.339, p < 10^-16^) between RNA and protein expression levels. While key biological processes, such as visual perception, were similarly regulated at both RNA and protein levels, others, including cell cycle processes and glycolysis, were predominantly regulated at the protein level. These findings highlight the value of integrating transcriptomics and proteomics for a more comprehensive understanding of the biological regulation, which is consistent with previous studies showing variable mRNA-protein correlations across different cancer types, pathways, and drug targets (35). A recent study across eight major cancer types reported a mean correlation between RNA and protein levels ranging between 0.4 and 0.6 depending on cancer type, with lung cancer having the highest and ovarian cancer having the lowest correlation (35). The discrepancies between transcriptomics and proteomics may also be explained by factors including protein and mRNA turnover/degradation, protein abundance and post-translational modifications. Technologies such as ribosome profiling or enrichment and quantification of newly synthesized proteins could help to explore this question in more detail.

Our analysis highlights that both transcriptomics and proteomics technology provide unique information and offer complementary insights. While proteomics allows direct analysis of the functional molecules in a biological system, RNA sequencing has the advantage of a higher molecular resolution with the ability to amplify transcripts with low abundance, detect transcripts from all protein coding genes as well as transcripts with regulatory functions (lncRNA, miRNA). The Human Protein Project has achieved remarkable progress, with protein expression data now available for 93% of the 19,778 predicted human proteins (36). However, 1,381 proteins still remain undetected, potentially due to their low abundance or challenges and limitations in mass spectrometric detection (36).

The tumor microenvironment (TME) is well known to modulate tumor progression, therapeutic response and clinical outcome of various malignancies (37). Here, we utilized transcriptome-based cell type deconvolution analysis and highly multiplexed spatially resolved single-cell proteomics using Imaging Mass Cytometry (IMC) to analyze retinoblastoma and adjacent retinal tissue. Our analysis revealed that the TME in retinoblastoma predominantly consists of multiple immune cell types, including CD4+ T cells and antigen presenting cells. The increased protein expression of CD68 in tumor-associated antigen presenting cells could indicate enhanced phagocytic activity. However, elevated CD68 expression is also linked to more aggressive tumors and a poor prognosis in various tumor types, including glioblastoma, kidney, hepatocellular, lung and other cancers (32). Finally, we identified two distinct subclusters of tumor cells: proliferating cells and CD44-positive tumor cells. The expression of CD44 may identify a population of cancer stem cells with potential involvement in metastatic spread, which is further supported by high protein levels of c-Myc in this cell population (33, 34). CD44 is also implicated in epithelial-to-mesenchymal transition and serves as a biomarker of poor prognosis in various cancer entities (33). Further research is needed to explore the prognostic significance and functional role of the identified cell populations in human retinoblastoma.

In search for new therapeutic strategies for children with retinoblastoma, the results of this study may help to inspire novel targeted therapies, including antibody-based interventions or immunotherapies targeting tumor cell specific epitopes (2). Furthermore, these results may enhance the molecular assessment of retinoblastoma in living patients. Since direct tumor biopsies are contraindicated in retinoblastoma patients due to risk of seeding, liquid biopsies from the aqueous humor in the anterior chamber of the eye emerge as a promising alternative approach for molecular analysis in living patients (2, 38–40). With our detailed understanding of transcriptomic and proteomic changes within the tumor, we can now explore the development of a proteomics-based liquid biopsy approach, focusing on proteins that exhibit alterations within the tumor and the adjacent fluid (38). As recently suggested, liquid biopsy-based monitoring may be part of future retinoblastoma treatment regimens (2), with potential clinical applications in diagnostics, prognostics, early detection of bilateral, trilateral or recurrent retinoblastoma, and monitoring of therapeutic response.

We acknowledge that this study is limited by its relatively small sample size, a consequence of the low incidence of retinoblastoma and the availability of effective eye globe-saving therapeutic modalities. Additionally, the long storage time may influence sequencing results. To account for this, we employed a specialized sequencing method that maintains high sequencing accuracy even after long term fixation (12). Future studies may utilize a similar multi-omics approach to a larger sample cohort with the goal to identify molecular tumor subclusters and to investigate prognostic associations. In our study, we dissected control tissue from the same tumor eyes, which has the strong advantage of having retinal tissue from the same children of the same age, otherwise not possible to obtain. However, we cannot fully exclude an influence of the tumor on control tissue, albeit microscopically not affected.

## Conclusions

In conclusion, this study integrated high-resolution proteomics, transcriptomics, and spatially resolved single-cell proteomics from the same samples to deliver a comprehensive proteotranscriptomic characterization of human retinoblastoma. The integration of these multiple omics technologies provides deeper insights into retinoblastoma biology that extend beyond the findings of single omics studies, potentially paving the way for novel targeted therapies for human retinoblastoma.

## Data Availability

The sequencing raw data generated in this study are available in the Gene Expression Omnibus Database: https://www.ncbi.nlm.nih.gov/geo/query/acc.cgi?acc=GSE276895. Processed and normalized data is provided in **Supplementary Table S3** (transcriptomics data) and **Supplementary Table S4** (proteomics data).

## Supplementary material

Supplementary Figure S1 Histology sections of all seven retinoblastoma eyes analyzed in this study. Hematoxylin and eosin stainings are shown. Abbreviations: C: cornea, L: lens, O: optic nerve, R: retina, S: sclera, T: tumor. The areas of interest (including their size) are highlighted in each section. Tumor and retinal control tissue could be isolated from patients 1-5, whereas only tumor tissue could be obtained from patients 6 and 7.

Supplementary Figure S2 Gene Set Enrichment Analysis (GSEA) in retinoblastoma. The top ten activated and suppressed biological processes selected by normalized enrichment score (NES) are shown in the dot plots. The NES is shown on the x-axis. The adjusted p-value of each term is indicated by color.

Supplementary Figure S3 Proteomic profile of human retinoblastoma. **(A)**: Unsupervised clustering using Principal Component Analysis (PCA) based on all 4,535 proteins detected using liquid chromatography-mass spectrometry. Each dot represents one sample. **(B)**: Heatmap visualizing differentially expressed proteins (DEP) between retinoblastoma and retinal control tissue (Definition of DEP: log2FC > 0.58 or < -0.58 and adjusted p-value < 0.05). Basic demographic data is shown at the top. Each column represents one sample and each row one DEP. The number of DEP is given within the heatmap. Time in formalin and age are given in years. The z-score represents a protein’s abundance in relation to its mean abundance by standard deviation units (red: upregulation, blue: downregulation). **(C, D)**: Gene ontology (GO) analysis of up- **(C)** and downregulated **(D)** proteins in retinoblastoma. The top ten enriched biological processes are shown in the dot plots. The size of the dots corresponds to the number of associated proteins (count). The adjusted p-value of each GO term is indicated by color. The gene ratio describes the ratio of the count to the number of all DEP.

Supplementary Figure S4 Transcriptome-based cell type deconvolution analysis in human retinoblastoma. The tool xCell uses gene expression profiles of 64 immune and stromal cell types to calculate cell type enrichment scores. The heatmap visualizes xCell enrichment scores of cell types which differed significantly between retinoblastoma and control tissue (p < 0.05, Mann–Whitney U test, and log2FC >1 or < -1). Each row represents one cell type, each column represents one sample. Rows are ordered according to the fold change of mean enrichment scores. Basic demographic data are shown above. Time in formalin and age are given in years.

## References

[1] Munier FL Beck-Popovic M Chantada GL Cobrinik D Kivela TT Lohmann D. Conservative management of retinoblastoma: Challenging orthodoxy without compromising the state of metastatic grace. “Alive, with good vision and no comorbidity. Prog Retin Eye Res. (2019) 73:100764. doi: 10.1016/j.preteyeres.2019.05.005 31173880

[2] Cobrinik D. Retinoblastoma origins and destinations. N Engl J Med. (2024) 390:1408–19. doi: 10.1056/NEJMra1803083 38631004

[3] Knudson AG Jr. Mutation and cancer: statistical study of retinoblastoma. Proc Natl Acad Sci U S A. (1971) 68:820–3. doi: 10.1073/pnas.68.4.820 PMC389051 5279523

[4] Elchuri SV Rajasekaran S Miles WO. RNA-sequencing of primary retinoblastoma tumors provides new insights and challenges into tumor development. Front Genet. (2018) 9:170. doi: 10.3389/fgene.2018.00170 29868118 PMC5966869

[5] Tang W Zhou M Dorsey TH Prieto DA Wang XW Ruppin E. Integrated proteotranscriptomics of breast cancer reveals globally increased protein-mRNA concordance associated with subtypes and survival. Genome Med. (2018) 10:94. doi: 10.1186/s13073-018-0602-x 30501643 PMC6276229

[6] Mun DG Bhin J Kim S Kim H Jung JH Jung Y. Proteogenomic characterization of human early-onset gastric cancer. Cancer Cell. (2019) 35:111–124.e110. doi: 10.1016/j.ccell.2018.12.003 30645970

[7] Ding N Zhang B Ying W Song J Feng L Zhang K. A time-resolved proteotranscriptomics atlas of the human placenta reveals pan-cancer immunomodulators. Signal Transduct Target Ther. (2020) 5:110. doi: 10.1038/s41392-020-00224-5 32606334 PMC7327038

[8] Wolf J Auw-Haedrich C Schlecht A Boneva S Mittelviefhaus H Lapp T. Transcriptional characterization of conjunctival melanoma identifies the cellular tumor microenvironment and prognostic gene signatures. Sci Rep. (2020) 10:17022. doi: 10.1038/s41598-020-72864-0 33046735 PMC7550331

[9] Wolf J Hajdu RI Boneva S Schlecht A Lapp T Wacker K. Characterization of the cellular microenvironment and novel specific biomarkers in pterygia using RNA sequencing. Front Med (Lausanne). (2021) 8:714458. doi: 10.3389/fmed.2021.714458 35174178 PMC8841401

[10] Wisner L Larsen B Maguire A. Enhancing tumor content through tumor macrodissection. J Vis Exp. (2022). 12(180)doi: 10.3791/62961 PMC10448995 35225270

[11] Wolf J Schlecht A Rosmus DD Boneva S Agostini H Schlunck G. Comparative transcriptome analysis of human and murine choroidal neovascularization identifies fibroblast growth factor inducible-14 as phylogenetically conserved mediator of neovascular age-related macular degeneration. Biochim Biophys Acta Mol Basis Dis. (2022) 1868:166340. doi: 10.1016/j.bbadis.2022.166340 35032596

[12] Boneva S Schlecht A Bohringer D Mittelviefhaus H Reinhard T Agostini H. 3’ MACE RNA-sequencing allows for transcriptome profiling in human tissue samples after long-term storage. Lab Invest. (2020) 100:1345–55. doi: 10.1038/s41374-020-0446-z PMC7498368 32467590

[13] Galaxy C. The Galaxy platform for accessible, reproducible and collaborative biomedical analyses: 2022 update. Nucleic Acids Res. (2022) 50:W345–351. doi: 10.1093/nar/gkac247 PMC9252830 35446428

[14] Martin G Wolf J Lapp T Agostini HT Schlunck G Auw-Hadrich C. Viral S protein histochemistry reveals few potential SARS-CoV-2 entry sites in human ocular tissues. Sci Rep. (2021) 11:19140. doi: 10.1038/s41598-021-98709-y 34580409 PMC8476534

[15] Dobin A Davis CA Schlesinger F Drenkow J Zaleski C Jha S. STAR: ultrafast universal RNA-seq aligner. Bioinformatics. (2013) 29:15–21. doi: 10.1093/bioinformatics/bts635 23104886 PMC3530905

[16] Liao Y Smyth GK Shi W. featureCounts: an efficient general purpose program for assigning sequence reads to genomic features. Bioinformatics. (2014) 30:923–30. doi: 10.1093/bioinformatics/btt656 24227677

[17] Cunningham F Allen JE Allen J Alvarez-Jarreta J Amode MR Armean IM. Ensembl 2022. Nucleic Acids Res. (2022) 50:D988–95. doi: 10.1093/nar/gkab1049 PMC8728283 34791404

[18] Love MI Huber W Anders S. Moderated estimation of fold change and dispersion for RNA-seq data with DESeq2. Genome Biol. (2014) 15:550. doi: 10.1186/s13059-014-0550-8 25516281 PMC4302049

[19] Gu Z Eils R Schlesner M. Complex heatmaps reveal patterns and correlations in multidimensional genomic data. Bioinformatics. (2016) 32:2847–9. doi: 10.1093/bioinformatics/btw313 27207943

[20] Wickham H. ggplot2: elegant graphics for data analysis. New York: Springer-Verlag (2016).

[21] Wu T Hu E Xu S Chen M Guo P Dai Z. clusterProfiler 4.0: A universal enrichment tool for interpreting omics data. Innovation (Camb). (2021) 2:100141. doi: 10.1016/j.xinn.2021.100141 34557778 PMC8454663

[22] Aran D Hu Z Butte AJ. xCell: digitally portraying the tissue cellular heterogeneity landscape. Genome Biol. (2017) 18:220. doi: 10.1186/s13059-017-1349-1 29141660 PMC5688663

[23] Wagner GP Kin K Lynch VJ. Measurement of mRNA abundance using RNA-seq data: RPKM measure is inconsistent among samples. Theory Biosci. (2012) 131:281–5. doi: 10.1007/s12064-012-0162-3 22872506

[24] Thompson A Schafer J Kuhn K Kienle S Schwarz J Schmidt G. Tandem mass tags: a novel quantification strategy for comparative analysis of complex protein mixtures by MS/MS. Anal Chem. (2003) 75:1895–904. doi: 10.1021/ac0262560 12713048

[25] Cox J Mann M. MaxQuant enables high peptide identification rates, individualized p.p.b.-range mass accuracies and proteome-wide protein quantification. Nat Biotechnol. (2008) 26:1367–72. doi: 10.1038/nbt.1511 19029910

[26] Tweedie S Braschi B Gray K Jones TEM Seal RL Yates B. Genenames.org: the HGNC and VGNC resources in 2021. Nucleic Acids Res. (2021) 49:D939–46. doi: 10.1093/nar/gkaa980 PMC7779007 33152070

[27] Boneva SK Wolf J Hajdu RI Prinz G Salie H Schlecht A. In-depth molecular characterization of neovascular membranes suggests a role for hyalocyte-to-myofibroblast transdifferentiation in proliferative diabetic retinopathy. Front Immunol. (2021) 12:757607. doi: 10.3389/fimmu.2021.757607 34795670 PMC8593213

[28] Windhager J Zanotelli VRT Schulz D Meyer L Daniel M Bodenmiller B. An end-to-end workflow for multiplexed image processing and analysis. Nat Protoc. (2023) 18:3565–613. doi: 10.1038/s41596-023-00881-0 37816904

[29] Carpenter AE Jones TR Lamprecht MR Clarke C Kang IH Friman O. CellProfiler: image analysis software for identifying and quantifying cell phenotypes. Genome Biol. (2006) 7:R100. doi: 10.1186/gb-2006-7-10-r100 17076895 PMC1794559

[30] Schapiro D Jackson HW Raghuraman S Fischer JR Zanotelli VRT Schulz D. histoCAT: analysis of cell phenotypes and interactions in multiplex image cytometry data. Nat Methods. (2017) 14:873–6. doi: 10.1038/nmeth.4391 PMC5617107 28783155

[31] Haghverdi L Lun ATL Morgan MD Marioni JC. Batch effects in single-cell RNA-sequencing data are corrected by matching mutual nearest neighbors. Nat Biotechnol. (2018) 36:421–7. doi: 10.1038/nbt.4091 PMC6152897 29608177

[32] Zhang J Li S Liu F Yang K. Role of CD68 in tumor immunity and prognosis prediction in pan-cancer. Sci Rep. (2022) 12:7844. doi: 10.1038/s41598-022-11503-2 35550532 PMC9098459

[33] Xu H Niu M Yuan X Wu K Liu A. CD44 as a tumor biomarker and therapeutic target. Exp Hematol Oncol. (2020) 9:36. doi: 10.1186/s40164-020-00192-0 33303029 PMC7727191

[34] Lawson DA Bhakta NR Kessenbrock K Prummel KD Yu Y Takai K. Single-cell analysis reveals a stem-cell program in human metastatic breast cancer cells. Nature. (2015) 526:131–5. doi: 10.1038/nature15260 PMC4648562 26416748

[35] Arad G Geiger T. Functional impact of protein-RNA variation in clinical cancer analyses. Mol Cell Proteomics. (2023) 22:100587. doi: 10.1016/j.mcpro.2023.100587 37290530 PMC10388586

[36] Omenn GS Lane L Overall CM Lindskog C Pineau C Packer NH. The 2023 report on the proteome from the HUPO human proteome project. J Proteome Res. (2024) 23:532–49. doi: 10.1021/acs.jproteome.3c00591 PMC11026053 38232391

[37] Quail DF Joyce JA. Microenvironmental regulation of tumor progression and metastasis. Nat Med. (2013) 19:1423–37. doi: 10.1038/nm.3394 PMC3954707 24202395

[38] Wolf J Rasmussen DK Sun YJ Vu JT Wang E Espinosa C. Liquid-biopsy proteomics combined with AI identifies cellular drivers of eye aging and disease in *vivo* . Cell. (2023) 186:4868–4884.e4812. doi: 10.1016/j.cell.2023.09.012 37863056 PMC10720485

[39] Berry JL Pike S Shah R Reid MW Peng CC Wang Y. Aqueous humor liquid biopsy as a companion diagnostic for retinoblastoma: implications for diagnosis, prognosis, and therapeutic options: five years of progress. Am J Ophthalmol. (2024) 263:188–205. doi: 10.1016/j.ajo.2023.11.020 38040321 PMC11148850

[40] Wolf J Franco JA Yip R Dabaja MZ Velez G Liu F. Liquid biopsy proteomics in ophthalmology. J Proteome Res. (2024) 23:511–22. doi: 10.1021/acs.jproteome.3c00756 PMC10845144 38171013

