# Supplementary figures and images for: A proteotranscriptomic approach to dissect the molecular landscape of human retinoblastoma
Source: Front Oncol. 2025 May 6;15:1571702. doi: 10.3389/fonc.2025.1571702 (PMC12088971; doi:10.3389/fonc.2025.1571702)

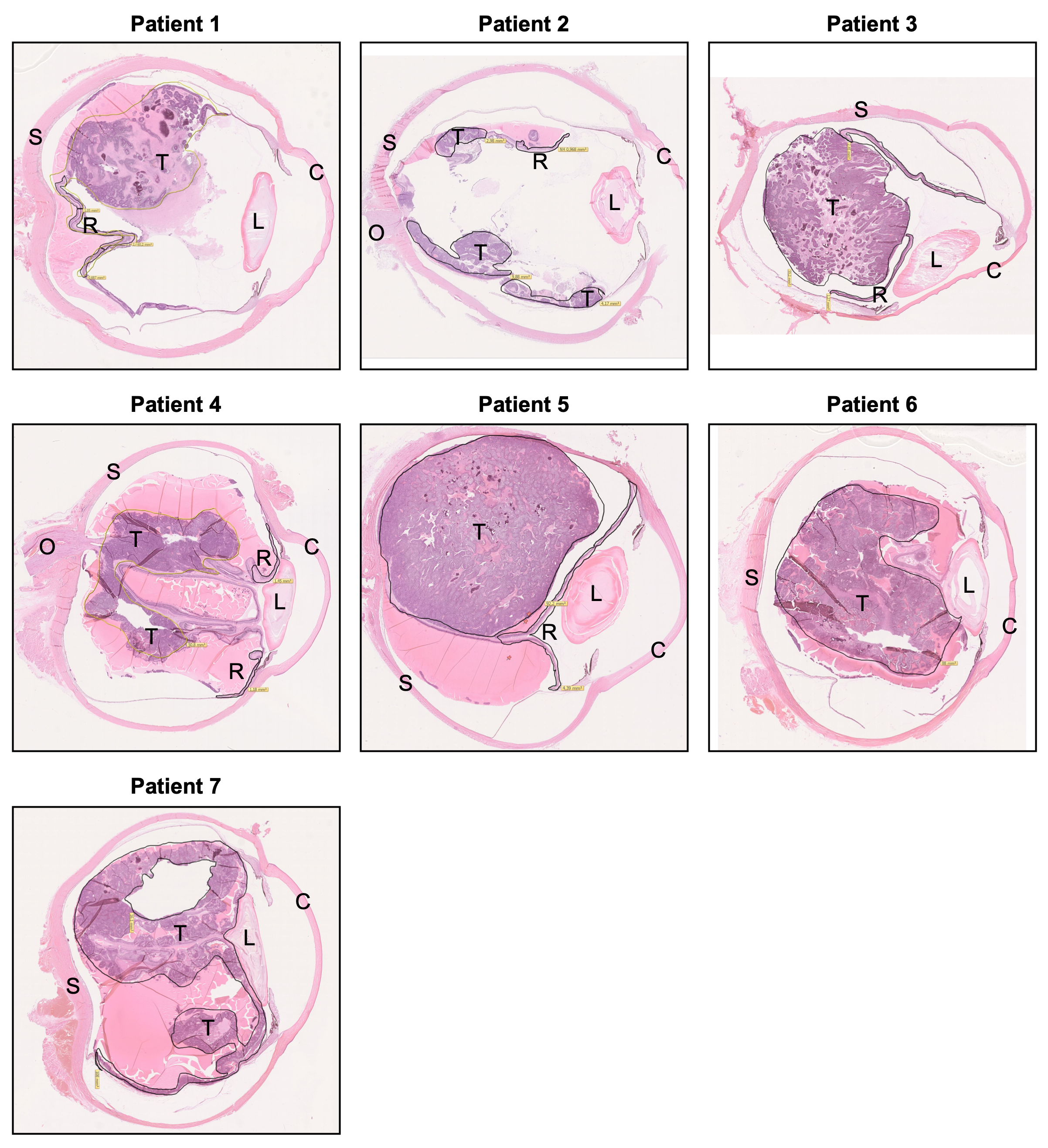

Supplement: Supplementary Figure S1 — Histology sections of all seven retinoblastoma eyes analyzed in this study. Hematoxylin and eosin stainings are shown. Abbreviations: C: cornea, L: lens, O: optic nerve, R: retina, S: sclera, T: tumor. The areas of interest (including their size) are highlighted in each section. Tumor and retinal control tissue could be isolated from patients 1-5, whereas only tumor tissue could be obtained from patients 6 and 7. [file Image1.tiff]

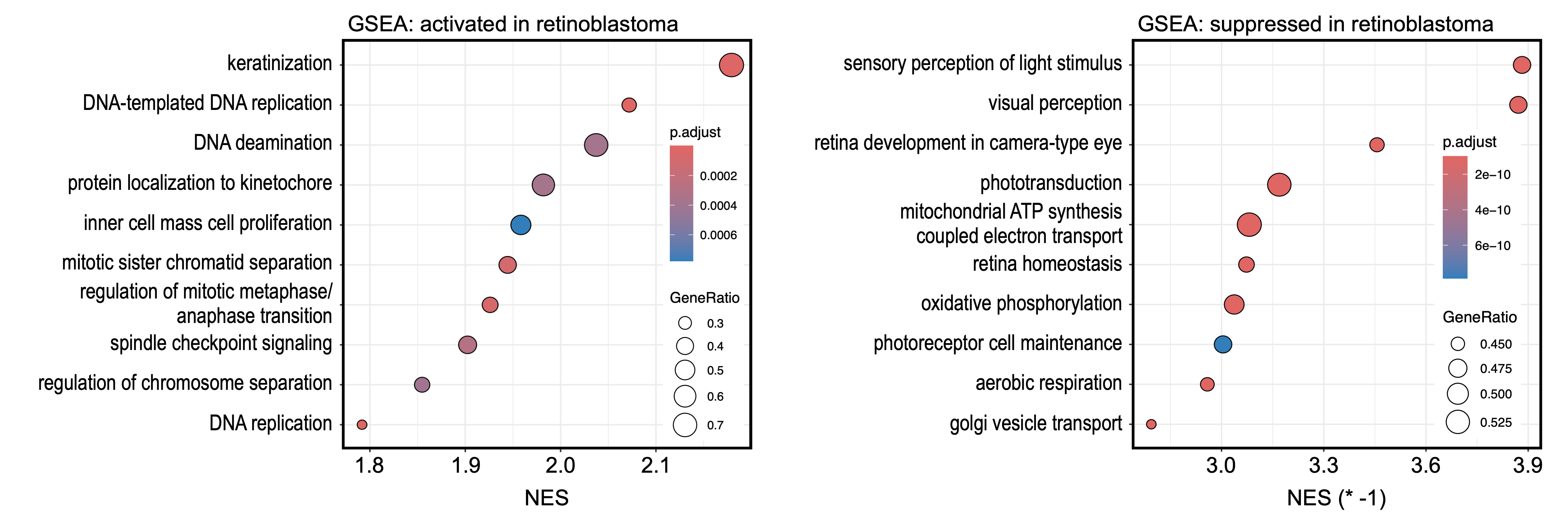

Supplement: Supplementary Figure S2 — Gene Set Enrichment Analysis (GSEA) in retinoblastoma. The top ten activated and suppressed biological processes selected by normalized enrichment score (NES) are shown in the dot plots. The NES is shown on the x-axis. The adjusted p-value of each term is indicated by color. [file Image2.tiff]

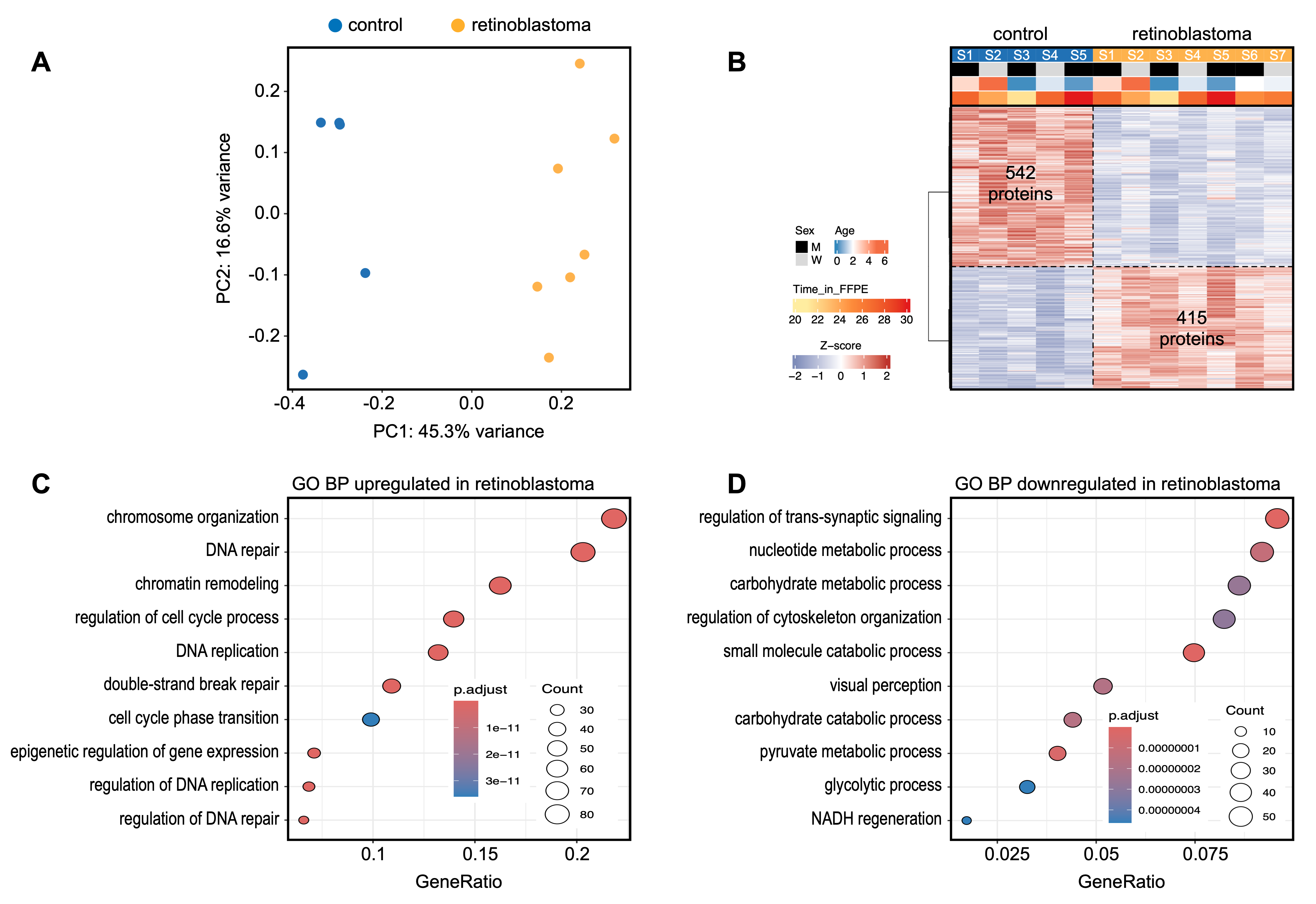

Supplement: Supplementary Figure S3 — Proteomic profile of human retinoblastoma. (A): Unsupervised clustering using Principal Component Analysis (PCA) based on all 4,535 proteins detected using liquid chromatography-mass spectrometry. Each dot represents one sample. (B): Heatmap visualizing differentially expressed proteins (DEP) between retinoblastoma and retinal control tissue (Definition of DEP: log2FC > 0.58 or < -0.58 and adjusted p-value < 0.05). Basic demographic data is shown at the top. Each column represents one sample and each row one DEP. The number of DEP is given within the heatmap. Time in formalin and age are given in years. The z-score represents a protein’s abundance in relation to its mean abundance by standard deviation units (red: upregulation, blue: downregulation). (C, D): Gene ontology (GO) analysis of up- (C) and downregulated (D) proteins in retinoblastoma. The top ten enriched biological processes are shown in the dot plots. The size of the dots corresponds to the number of associated proteins (count). The adjusted p-value of each GO term is indicated by color. The gene ratio describes the ratio of the count to the number of all DEP. [file Image3.tiff]

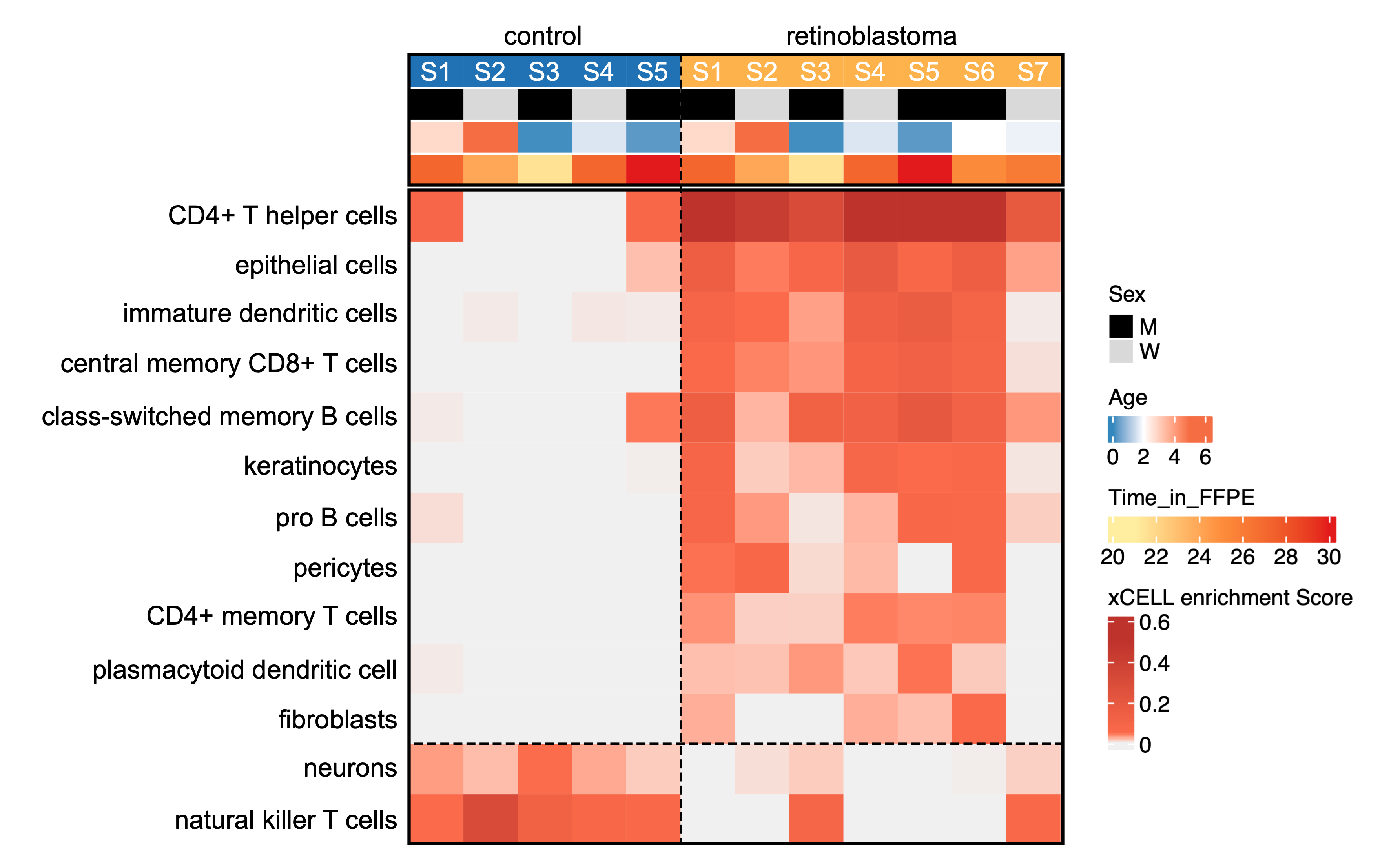

Supplement: Supplementary Figure S4 — Transcriptome-based cell type deconvolution analysis in human retinoblastoma. The tool xCell uses gene expression profiles of 64 immune and stromal cell types to calculate cell type enrichment scores. The heatmap visualizes xCell enrichment scores of cell types which differed significantly between retinoblastoma and control tissue (p < 0.05, Mann–Whitney U test, and log2FC >1 or < -1). Each row represents one cell type, each column represents one sample. Rows are ordered according to the fold change of mean enrichment scores. Basic demographic data are shown above. Time in formalin and age are given in years. [file Image4.tiff]
