# Supplementary material for: A proteotranscriptomic approach to dissect the molecular landscape of human retinoblastoma
Source: Front Oncol. 2025 May 6;15:1571702. doi: 10.3389/fonc.2025.1571702 (PMC12088971; doi:10.3389/fonc.2025.1571702)
Supplement: Supplementary file 7 [file DataSheet1.pdf]

Supplement Tables

Table S1: Patient characteristics and analyzed samples. RNA seq: RNA sequencing, LC-MS/MS: Liquid chromatography-mass spectrometry, IMC: Imaging Mass Cytometry. In bilateral cases, the enucleated eye was analyzed. ICRB: International Classification of Retinoblastoma.

	Patient	Age (years)	Sex	ICRB Stage (Philadelphia version)	Genetics	Histology	Uni-/bilateral	Follow-up (time after enucleation)	Tissues analyzed	Sample	Omics analyses		
											RNA seq	LC-MS/MS	IMC
1.	Patient 1	2.8	Male	D/E	NA	NA	Unilateral	Cancer-free (20 years)	Retinoblastoma	Retinoblastoma_S1	Yes	Yes	No
2.									Control	Control_S1	Yes	Yes	Yes
3.	Patient 2	5.1	Female	D	Constitutional heterozygosity Rb1.20, Rbi2	Moderate to well-differentiated	Unilateral	Cancer-free (2.3 years)	Retinoblastoma	Retinoblastoma_S2	Yes	Yes	Yes
4.									Control	Control_S2	Yes	Yes	No
5.	Patient 3	0.1	Male	D	NA	Well-differentiated	Unilateral	Cancer-free (2 years)	Retinoblastoma	Retinoblastoma_S3	Yes	Yes	Yes
6.									Control	Control_S3	Yes	Yes	Yes
7.	Patient 4	1.6	Female	E	NA	Well-differentiated	Unilateral	Cancer-free (10 years)	Retinoblastoma	Retinoblastoma_S4	Yes	Yes	Yes
8.									Control	Control_S4	Yes	Yes	Yes
9.	Patient 5	0.3	Male	E	NA	Well-differentiated	Unilateral	Cancer-free (12 years)	Retinoblastoma	Retinoblastoma_S5	Yes	Yes	Yes
10.									Control	Control_S5	Yes	Yes	Yes
11.	Patient 6	2.0	Male	D	NA	Undifferentiated	Bilateral	Fellow eye (11 months)	Retinoblastoma	Retinoblastoma_S6	Yes	Yes	Yes
12.	Patient 7	1.8	Female	E	Parents denied genetic test	Moderate to well-differentiated	Bilateral	Fellow eye (6 months) Cancer-free (10 years)	Retinoblastoma	Retinoblastoma_S7	Yes	Yes	Yes

Table S2: Antibodies, clones, conjugated metals, and applied concentrations for Imaging Mass Cytometry analysis.
Catalogue numbers starting with “ab” are from Abcam, all other antibodies are from Fluidigm.

Target	Clone	Metal	Concentration	Catalogue No.
aSMA	1A4	141Pr	1:800	3141017D
EGFR	D38B1	142Nd	1:800	3142013D
p53	DO-7	143Nd	1:400	3143026D
Rb	EPR17512	145Nd	1:200	ab218526
Bcl2	EPR17509	146Nd	1:400	3146019D
CD163	EDHu1	147Sm	1:800	3147021D
Pan-keratin	C11	148Nd	1:800	3148020D
CD11b	EPR1344	149Sm	1:800	3149028D
CD31	EPR3094	151Eu	1:800	3151025D
CD45	D9M8I	152Sm	1:800	3152018D
CD44	IM7	153Eu	1:800	3153029D
Beta-actin	2F1-1	154Sm	1:800	3154021D
FoxP3	PCH101	155Gd	1:800	3155018D
CD4	Epr6855	156Gd	1:800	3156033D
E-cadherin	24E10	158Gd	1:800	3158029D
CD68	KP1	159 Tb	1:800	3159035D
CD274/PD-L1	SP142	160Gd	1:200	ab236238
CD20	H1	161Dy	1:200	3161029D
CD8a	C8/144B	162Dy	1:800	3162034D
VEGF	G153-694	163Dy	1:100	3163028D
Arginase-1	D4E3M	164Dy	1:100	3164027D
PD1	EPR4877(2)	165Ho	1:400	3165039D
CD74	LN2	166Er	1:800	3166025D
c-Met	D1C2	167Er	1:50	3167020D
Ki-67	B56	168Er	1:800	3168022D
Collagen type I	Poly	169Tm	1:800	3169023D
CD3	Poly	170Er	1:200	3170019D
Histone H3	D1H2	171Yb	1:800	3171022D
Caspase3	5A1E	172Yb	1:400	3172027D
CD276/B7-H3	Poly	173Yb	1:800	3173014D
HLA-DR	LN3	174Yb	1:800	3174025D
Pan-Actin	D18C11	175Lu	1:800	3175032D
c-Myc	9E10	176Yb	1:50	3176012B
DNA	Iridium	191/193 Ir	1:2000	201192B
